# Complications, Functional Recovery, and Quality of Life in Breast Cancer Patients Undergoing Axillary Surgery

**DOI:** 10.1155/tbj/1469203

**Published:** 2026-07-08

**Authors:** Monica Pellegrino, Francesco Ioppolo, Monia Ranalli, Michelangelo Miccini, Francesca La Rovere, Stefania Lancia, Stefano Avenia, Paolo Izzo, Giulia Montagner, Giuliano D’Onghia, Andrea Polistena

**Affiliations:** ^1^ Department of Surgery “Pietro Valdoni”, Policlinico “Umberto I”, Sapienza” University of Rome, Rome, 00128, Italy; ^2^ Department of Physical Medicine and Rehabilitation, Policlinico “Umberto I”, “Sapienza” University of Rome, Rome, 00128, Italy, uniroma1.it; ^3^ Department of Statistical Sciences, Policlinico “Umberto I”, “Sapienza” University of Rome, Rome, 00128, Italy, uniroma1.it; ^4^ Department of Medicine and Surgery, University of Perugia, Perugia, 06100, Italy, unipg.it

**Keywords:** complications, DASH, de-escalation of axillary surgery, disability, rehabilitation

## Abstract

**Background:**

Survivorship considerations have gained increasing importance in patients with early breast cancer. Every surgical procedure poses a risk of complications and a potential negative impact on patient‐reported outcomes (PROs), thereby driving growing interest in de‐escalation strategies in breast cancer surgery. In this context, we aimed to assess complication rates, the potential role of physiatric rehabilitation in managing these complications, and the impact of axillary surgery on PROs.

**Methods:**

This retrospective single‐center cohort study included breast cancer patients who underwent surgery between January 2022 and March 2023 at the Breast Unit of Policlinico Umberto I, Rome. Among 164 patients operated on during the study period, 71 who underwent axillary surgery and received postoperative physiatric evaluation according to the institutional care pathway (PDTA) were included. The Disability of the Arm, Shoulder and Hand (DASH) questionnaire was administered to evaluate PROs.

**Results:**

Among the included patients (*n* = 71), SLNB was performed in 71.83% and ALND in 21.13%. A total of 34/71 patients (47.89%) developed complications requiring rehabilitative treatment and were classified as cases, while the remaining patients constituted the control group. Overall, shoulder painful mobility limitation (56.34%) (SPML), motor deficit (54.93%) (MD), and sensory deficit (46.48%) (SD) were the most frequent complications, whereas lymphedema (LE) and scapular winging (SW) occurred in 4.23% each. More extensive axillary procedures were associated with a higher number of lymph nodes removed (*p* < 0.0001). Cases had significantly more lymph nodes removed than controls (*p* = 0.0003). Patients requiring rehabilitation were younger on average than controls. Recovery time differed significantly between cases and controls (*p* < 0.0001), with most patients recovering within 3 months from the first physiatric visit. DASH scores were significantly worse in patients requiring rehabilitative treatment than in controls (*p* < 0.0001). The mean DASH score was 14%. Higher DASH values were associated with postoperative complications requiring rehabilitation and delayed or absent recovery, particularly SPML, DM, DS, and SW.

**Conclusions:**

Functional impairment of the upper limb remained frequent and may affect patients’ daily activities. These findings support the importance of integrating surgical management with early physiatric evaluation and rehabilitation to optimize functional recovery. Systematic assessment using patient‐reported outcome measures (PROMs), such as the DASH questionnaire, may help identify disability early and support patient follow‐up.

## 1. Introduction

Breast cancer (BC) is the most diagnosed malignancy in women, representing the most frequently diagnosed tumor across all age groups [1]. Integrated care through multidisciplinary meetings (MDM) in Breast Units is crucial, providing patients’ comprehensive support from screening to surgical treatment through rehabilitation at every disease stage with the aim of intercepting and preventing the onset of structural and functional alterations to promote an early return to daily life activities and maximum psychophysical functional recovery [2, 3]. Therefore, “rehabilitative care” by the physiatrist specialist is necessary through the formulation of an Individual Rehabilitation Project (IRP) [4].

Axillary sentinel lymph node biopsy (SLNB) and axillary lymph node dissection (ALND) represent standard procedures in BC treatment. SLNB is the gold standard for axillary staging patients with clinical stages I–II of BC and clinically negative lymph nodes or clinically suspicious lymph nodes but with subsequent negative needle biopsy or fine‐needle aspiration cytology (FNAC). ALND is recommended in the presence of clinically pathological axillary lymph nodes confirmed by preoperative FNAC, in cases where a sentinel lymph node is not found, in T4 tumors and inflammatory carcinoma, and in case of positive lymph node after primary SLNB after discussion in MDM [5]. Both procedures are associated with surgical complications and potential residual morbidity, mostly represented by minor drawbacks, such as seromas (SE), sensory deficit (SD), such as paresthesia, motor deficit (MD), scar complications (SC), and axillary web syndrome (AWS), and major events, such as lymphedema (LE), scapular winging (SW), and shoulder painful motility limitation (SPML). ALND is more often followed by these major complications, resulting in a high morbidity burden [6].

These adverse effects interfere with daily activities, are distressing, impair quality of life (QoL), and are costly in terms of rehabilitation treatments, as they are often permanent, and symptom relief is difficult. It is precisely from this consideration that, in recent years, a paradigm shift has been underway in axillary management, also calling into question the role of ALND for women with early‐stage BC and one or two metastases on SLNB, with a general trend toward evaluating the application of progressive de‐escalation in axillary surgery to potentially reduce treatment‐related morbidity without compromising oncologic outcomes [7].

The aim of this study was to evaluate the prevalence of postoperative complications following axillary surgery for BC in our clinical practice and, above all, to analyze the subsequent need for a specific rehabilitation program through an integrated surgical and physiatric evaluation, observing postoperative functional recovery and the patient’s perceived disability using the DASH questionnaire.

## 2. Methods

### 2.1. Study Design and Endpoints

In our institution, Policlinico Umberto I, Academic Hospital, Sapienza University of Rome, Italy, all BC patients are referred to the Breast Unit that offers the care of patients through MDM for the entire course of care and assistance. Since January 2022, a Diagnostic Therapeutic Care Pathway (PDTA) with physiatric assessment has been implemented to provide PRI based on postoperative morbidity severity, meeting EUSOMA quality standards for BC center [8–10].

This single‐center retrospective observational cohort study was reported in accordance with the Strengthening the Reporting of Observational Studies in Epidemiology (STROBE) guidelines [11], and the completed STROBE checklist was submitted as supporting information. The study was conducted in accordance with the Declaration of Helsinki, but the protocol was not submitted to the evaluation of the local ethics committee or registered as a clinical trial due to the retrospective design of the research. All patients gave their informed consent to the use of their clinical data for research purposes at the time of surgery. This research received no external funding.

In particular, the primary endpoint was to study the prevalence of complications in the selected population, associating them with the type of surgical treatment received, lymph node status, disease stage (TNM), and patient characteristics (age, BMI); the secondary endpoint was the evaluation of the presence/absence of functional recovery and its timing in relation to the first physiatric visit and after any physiatric and physiotherapeutic treatment performed, considering the disability experienced by patients quantified through the Disability of the Arm, Shoulder and Hand (DASH) questionnaire [12, 13] in order to assess the implications on QoL.

### 2.2. Observation Period

All patients examined were operated on for BC by the same surgical team (AP and MM), with standard surgical techniques, during the period between January 2022 and March 2023 (15 months).

### 2.3. Patient Characteristics

We retrospectively studied 164 BC patients operated on during the observation period, and we identified 71 who underwent breast surgery together with an axillary surgical procedure (SLNB or ALND, or ALND after positive SLNB) and who, according to the PDTA, underwent a physiatric clinical evaluation after surgery.

The study population was subsequently stratified into two cohorts. The “case” cohort encompassed 34 patients who manifested complications necessitating rehabilitative intervention. Conversely, the “control” cohort comprised 37 patients who did not develop complications severe enough to necessitate rehabilitation.

Inclusion criteria considered were as follows: patients aged ≥ 18 years, who have undergone any type of axillary surgery as stated above and have on biopsy‐proven BC with indication to staging SLNB or ALND according to guidelines. Instead, the patients not receiving axillary surgery or those declining the prescription of a physiatric consultation after surgery or those lost to follow‐up, thus with unavailable data regarding axillary functional outcome, were excluded. Medical records in the observational period were collected from our database and analyzed anonymously.

### 2.4. Preoperative Workup

Axillary preoperative staging was performed on all patients using ultrasound (US) as the first‐line imaging modality. Those with suspicious lymph nodes underwent FNAC to check for metastases [14]. Furthermore, patients with evidence or suspicion of locally advanced cancer or of multifocal tumors, and all patients with indication to neoadjuvant treatment underwent breast contrast‐enhanced magnetic resonance imaging (MRI) with imaging detection of pathological axillary lymph nodes. This study was conducted in a non‐neoadjuvant setting; therefore, targeted axillary dissection (TAD) was not included as an axillary staging method. Moreover, in BC patients converting from clinically positive (cN+) to negative (ycN0) lymph node status after neoadjuvant chemotherapy (NACT), the different axillary surgical staging procedures are currently under debate [15, 16]: The ongoing AXSANA study [17] showed that less invasive surgical staging procedures are associated with a low axillary recurrence rate, not inferior to ALND after 3 years. These findings reinforce efforts to minimize surgical morbidity without compromising oncologic outcomes [18], but further follow‐up is necessary to assess long‐term results and the effect of differences in staging accuracy on BC‐specific survival [19].

Preoperative workup included blood tests, ECG, chest X‐ray/computed tomography scan if required, cardiac risk, and anesthesiologic assessment. The surgical procedure for SLNB included preoperative (1 to 24 h before surgery) peritumoral injection of radioactive colloid (99 m Tc sulfur colloid) with the amount of colloid injected varying, depending on the projected time to surgery. Then, the sentinel lymph node is identified by the radioactivity counts emitted from the node and recorded by a handheld gamma probe (Neoprobe, Devicor Medical Italy Srl) [20, 21].

### 2.5. Surgical Procedure

Regarding the surgical practice of SLNB, the patient was positioned supine on the operating table and rotated about 20° away from the operator, with the ipsilateral arm extended and abducted at 90°. To avoid possible brachial plexus injuries, any hyperextension maneuver of the limb should be avoided. A skin incision of about 2 cm is made along the ideal incision line for axillary dissection, which is normally performed along a skin fold. This allows, by extending the incision laterally and medially, to convert the biopsy procedure into a dissection if necessary, without having to make additional incisions [22] and reducing the possible occurrence of AWS. A drain was not placed in the axilla. The sentinel node was submitted for definitive histological evaluation according to the recommendations of the Italian Pathology and Cytology Association (SIAPEC) and of the Italian Breast Pathologist Association (GIPAM) [23].

The ALND involves the removal of at least 10 lymph nodes for accurate pathological evaluation of the axilla [24]. ALND was performed in continuity with breast excision only for tumors in upper‐outer quadrants (QSE), avoiding limb root incisions to prevent functional limitations to AWS. For tumors in other locations, separate axillary incisions were used. ALND should include tissue superiorly to the level of the axillary vein, medially to the chest wall, laterally to the latissimus dorsi muscle, inferiorly to the interdigitation of the latissimus dorsi and serratus anterior muscles, and posteriorly to the subscapularis muscle. During dissection, particular care should be taken to avoid excessive skeletonization of the long thoracic nerve, which adds nothing to the radicality of the procedure, as well as detaching the nerve trunk from the thoracic wall, which increases the risk of stretching the terminal branches, and excessively coagulating small vessels in its proximity, as its compromise increases the probability of onset of a MD in the serratus anterior muscle innervated by it, a major complication known as SW [25].

The intercostal–brachial nerve, running medial to lateral, should be preserved during the dissection, and it is considered a standard in axillary surgery according to the conclusions reached during the St. Gallen Consensus Conference in 2021 [2]. All lymphatic tissue is dissected by Harmonic Focus (Ethicon, Johnson & Johnson), and clips or tie application is used close to vessels or nerves [22]. A Jackson–Pratt drain is brought out from the axilla.

### 2.6. Postoperative Workup and Physiatric Intervention

A postoperative surgical clinical evaluation was provided to all patients who underwent axillary surgery to exclude early complications within 2 weeks after the surgery and investigate the possible presence of SC, AWS, and SE [26, 27]. Then, they were subjected to physiatric clinical evaluation at 1, 3, and 6 months after the surgery to detect any late‐onset complications, such as SMPL, MD, SD, LE, and SW complications.

The rehabilitative treatment for AWS involved gentle and progressive stretching maneuvers of the upper limb to promote elongation and prevent functional limitations, associated with manual lymphatic drainage (MLD) limited to the cording in a centripetal distoproximal direction up to the axillary hollow. Patient education was always indicated to introduce, within the self‐care and self‐treatment program, specific maneuvers for cording, if present. This facilitated the functional recovery of the upper limb in a short time [28].

If collections, such as SE, are present, antibiotic therapy was administered, and if the volume exceeds 40 cc, percutaneous aspiration and compressive bandaging were performed, while specific rehabilitative intervention was contraindicated. Patients experienced SD in axilla and posterior–medial arm, including hypoesthesia, anesthesia, and paresthesias in the intercostobrachial nerve distribution. Treatment primarily involves reassurance about recovery, with pain medication rarely necessary.

During the clinical examination, we investigated the SW, a condition in which the medial border and inferior angle of the scapula protrude prominently from the chest wall, with a deficit in forward flexion and abduction of the arm beyond 100° with the elbow extended due to a long thoracic nerve deficit and impairment of the serratus anterior muscle function.

Therefore, the physiokinesitherapy (FKT) with neuromuscular facilitation techniques was performed to promote the coaptation of the scapula to the chest wall, compensated by the other muscles of the scapulohumeral girdle and the trapezius [29, 30]. Postoperatively, patients developed protective posture with an elevated, internally rotated, adducted arm and dorsal hyperkyphosis, limiting shoulder ROM (range of motion). Physiotherapy provided comprehensive rehabilitation targeting upper limbs and cervico‐dorsal spine to correct posture, prevent permanent issues, relieve pain, and restore proper function.

Alongside physiotherapy, mesotherapy (MT) was performed to control SMPL by injecting microdeposit, an anti‐inflammatory drug in the skin dermis, slowing down the kinetics, absorption, and prolonging the local mechanism of action. It was applied successfully as a treatment for some forms of localized painful syndromes and SC, softening their fibrous tissue and restoring normal nerve conduction in the affected area [31].

Finally, LE is a major complication of axillary surgery caused by lymphatic disruption and lymph node removal.

The treatment program of “Complex Decongestive Therapy” (CDT) is involved in two phases [29]: The first one with MLD serves to improve the filling of primary cutaneous lymphatic vessels, and it was performed weekly, and, after or concurrently, the second one with elastic compression multilayer bandaging (CB) that increased interstitial pressure and increased lymphatic flow, resulting in a reduction of LE volume.

It was done by placing a tubular German cotton mesh on the arm to protect the skin, followed by three short‐stretch bandages up to 4 layers, thus obtaining compression of the limb reaching a pressure of about 20–30 mmHg. The bandages were then removed the next day, to be reapplied until the best possible decongestion was achieved. Once a decongestion plateau was reached, the maintenance phase began, which aimed to preserve and optimize results and included the use of an elastic brace/stocking. Patients had to understand LE as a chronic condition, not just a symptom, with brace usage varying based on the extent of lymphatic insufficiency and patient compliance with possible intermittent wear focused on risk activities [32].

All our enrolled patients were administered the DASH questionnaire through a telephone interview based on self‐assessment. The DASH questionnaire is recommended to evaluate functional activity profiles in patients with upper extremity disorders and describe the disability experienced by patients diagnosed with BC who have undergone surgical intervention [33]. The assessment uses a 38‐question scale divided into three modules: a mandatory 30‐question main module about daily activities, and two optional 4‐question modules for work and sports/recreation activities. In particular, the main module mandatory consists of 16 questions related to the functionality of the shoulder, arm, and hand that investigate daily life activities (writing, turning a key, opening a jar…), four questions related to recreational activities (playing cards, knitting, and playing frisbee), three questions related to the patient’s perception of disability in work and social activities, five questions that investigate present symptoms (pain, tingling, weakness, and stiffness), one question related to difficulty sleeping due to pain, and one question related to loss of confidence due to the problem. All questions use a five‐point Likert scale (1 = *no difficulty* to 5 = *unable*). Scores are calculated as ([sum of responses/number of completed responses]−1) × 25, ranging from 0% to 100%, with higher scores indicating greater disability. Only the main module was used in this evaluation.

### 2.7. Statistical Analysis

First, we analyzed the data through the main descriptive statistics. We summarized the quantitative variables by means and standard deviations and the qualitative variables by percentage frequencies.

Then, the data were grouped and summarized in simple and double frequency tables to describe the main characteristics of the case/control groups to study the prevalence of complications during the aforementioned observation period. Subsequently, to evaluate correlations between variables, statistical tests, such as the *T*‐test (also known as Student’s *t*‐test) and the chi‐square test, were used for quantitative and qualitative variables, respectively. Specifically, the former was used to associate quantitative variables (e.g., DASH scores) in the two groups (e.g., cases vs. controls); the latter was used to verify the independence between qualitative variables, using contingency tables (e.g., various complications related with respect to the type of axillary treatment). In cases where the variable had more than two categories, the ANOVA test was applied. For all tests, a *p*‐value < 0.05 was considered statistically significant. The data were analyzed using the statistical software R (free open source). No missing data were observed for any of the variables included in the analysis.

## 3. Results

From the initial descriptive analysis, it emerged that among the 71 observed patients, 53.52% underwent a breast‐conserving surgery procedure, primarily followed by SLNB. The remaining 46.48% underwent a mastectomy, followed in equal percentages by either SLNB or ALND. The axillary intervention was performed on all patients: 71.83% underwent SLNB, 21.13% ALND, and 7.04% underwent ALND after SLNB.

The mean age of the patients was 61 years (±13.71), and the average BMI was 25 (±4.68). Based on final pathological staging (pTNM), the majority of patients showed stage IA (T1N0M0) and stage IIA (T0/T1/T2 N1M0) disease, corresponding overall to an early stage. Specifically, among patients who underwent SLNB, the majority (40%) presented stage IA disease, whereas among those who underwent ALND, stage IIIA disease was observed in 7.14% of this subgroup.

The complications observed among the 71 selected patients included LE, SC, SE, SPML, DM, DS, and SW, with rates of 4.23%, 9.86%, 2.82%, 56.34%, 54.93%, 46.48%, and 4.23%, respectively.

**FIGURE 1:**
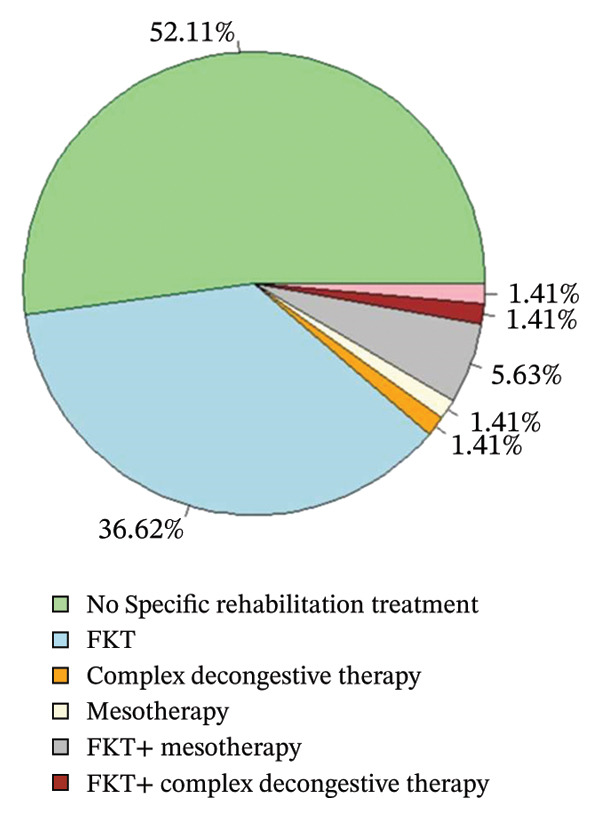
Physiatric/physiotherapy treatment. FKT = physiokinesitherapy.

About 38.03% of patients presented with both DM and DS. Conversely, 34.72% of patients (*n* = 25/71) did not develop any complications. Recalling that the 71 patients were selected based on their attendance at the first physiatric visit, we observed that more than half, 52.11% (*n* = 37/71), did not require subsequent specific rehabilitation intervention and constituted the control group. Overall, 34 of the 71 patients (47.89%) developed complications requiring rehabilitative treatment and were therefore classified as cases. Among those who continued the therapeutic process, the majority, 36.62%, underwent FKT only, 1.41% CDT, 1.41% MT, while 5.63% received a combined treatment of FKT and MT, and 1.41% received a combined treatment of FKT and CDT (Figure 1).

**FIGURE 2:**
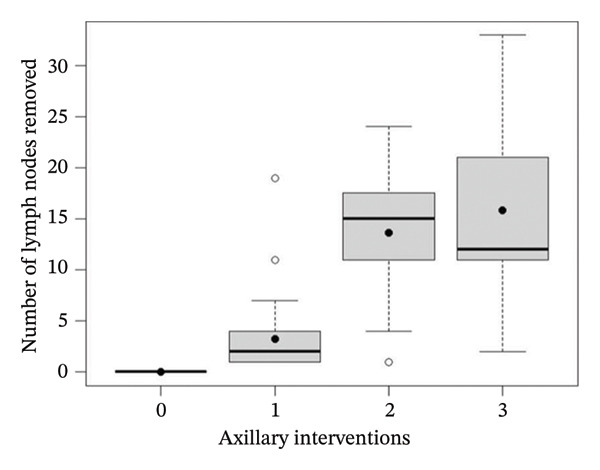
Boxplot 1 represents the distributions of the number of lymph nodes removed conditionally on the type of surgery. The differences between intervention 2 (ALND) and 3 (ALND after SLNB) compared to 0 (no intervention) were statistically significant. Number 1 was SLNB. ALND = axillary lymph node dissection. SLNB = axillary sentinel lymph node biopsy.

Then, the characteristics of lymph node variables were evaluated in relation to the type of surgical treatment received. In particular, the mean of total lymph nodes removed conditional on axillary intervention is strongly statistically different (*p* ≤ 0.0001) with values of 3.37 for the SLNB procedure, 13.66 for ALND, and 15.80 for ALND after SLNB. Indeed, we can see from the boxplots how the average number of removed lymph nodes increases in relation to the more extensive type of intervention. In particular, the differences between intervention 2 (ALND) and 3 (ALND after SLNB) compared to no intervention were statistically significant (Figure 2: Boxplot 1).

We then correlated the onset of complications with the different surgical procedures performed, observing a statistically significant association between SC and axillary interventions (*p* = 0.0442), and between DS and DM with breast surgery and axillary interventions (DS: *p* = 0.0382, *p* = 0.00040; DM: *p* = 0.0278, *p* = 0.0170). From the correlation between complications and lymph node variables, the mean numbers of total lymph nodes removed were statistically different conditional on DM (*p* = 0.0010) and DS (*p* = 0.0124), with average values of about 8 total lymph nodes removed in those presenting these complications.

From the evaluation of patient characteristics in relation to complications, we obtained a statistically significant correlation between SC and BMI (*p* = 0.0414), with the presence of SC in those with a lower BMI (mean of 22.55) compared to those with a higher average BMI (25.20). Evaluating the stage of disease, a statistically significant association emerged with DM (*p* = 0.0072) and DS (*p* = 0.0007), where the presence of the latter appeared to be more prevalent in stages higher than IIB.

The second part of the statistical analysis was applied to the cases and controls defined above. The mean age value in the two groups was borderline statistically significant, with an average age of 58 years in the cases and 64 in the controls (*t* = −1.872 *p* = 0.0654), whereas the BMI did not show a statistically significant difference and showed an average of about 24 in both groups (*t* = −0.090 *p* = 0.9282). Furthermore, the two groups did not differ significantly in terms of disease stage either.

However, in relation to lymph nodes, they were statistically different with respect to the total lymph nodes removed (*p* = 0.0003), with a conditional mean of 9.26 compared to 3.40 in the case/controls groups, respectively. A statistically significant dependence also emerged in the distribution between the types of axillary intervention over the two groups. In particular, SLNB intervention prevailed in the controls, while in the cases, more ALND and ALND after SLNB interventions were performed. A comparison was made between these interventions, and a statistically different dependence (*p* = 0.0119) was found between the proportions of ALND and ALND after SLNB interventions (0.428), conditional on cases and controls.

As regards LE, SC, SE, SPML, DM, DS, and SW, the respective proportions were analyzed in the case and control groups, and a statistical association emerged for SPML (*p* ≤ 0.0001), DM (*p* ≤ 0.0001), and DS (*p* = 0.0014). In particular, these were found to be more prevalent in the cases compared to the controls, at 91%, 82%, and 64%, respectively. For AWS, a significant association emerged (*p* = 0.0052), with statistically different relative proportions (*p* = 0.0280) of the type of axillary intervention in relation to the presence of AWS in cases and controls. Indeed, a statistical dependence was found between SLNB, ALND, and ALND after SLNB in the two groups, with a proportionally higher development of the AWS complication in the case group among the patients who underwent SLNB.

Regarding the complications of LE and SW, the small number of patients (3 and 3, respectively) did not allow for a similar evaluation of a possible correlation. The three patients who presented with LE underwent SLNB, ALND, and ALND after SLNB, respectively. Of the three patients who presented with SW, 2 underwent ALND and 1 underwent ALND after SLNB.

On the other hand, recovering (present/absent) had no different effect on the two groups (*p* = 0.1361). However, it was possible to observe that in the cases, we had a recovery in 67.39% of patients, while in the controls, it was present only for 23.91%.

Moreover, overall, considering the total number of patients (*n* = 71), in those who developed complications (*n* = 46), excluding the asymptomatic ones (*n* = 25), the recorded recovery was 91.3%. Concerning recovery times, they were found to have a different effect over the two groups (*p* ≤ 0.0001). Proportionally, in the two groups, we observed a greater recovery time within 3 months from the first physiatric visit (*p* = 0.0141) (Table 1).

**TABLE 1:** Evaluation of presence/absence of recovery, timing, and DASH scores in case/control groups.

	Recover no		Recover yes		*p*‐value
0	4.35%		23.91%		0.1361
1	4.35%		67.39%		
Not asymptomatic	8.7%		91.3%		
	Dash				*p*‐value
0	4.53%				< 0.0001
1	24.95%				
Recovery times	0	1	2	4	*p*‐value
0	4.44%	20%	2.22%	2.22%	< 0.0001
1	4.44%	17.78%	26.67%	22.22%	
	0.50	0.52	0.07	0.09	0.01412

*Note:* 0 = Controls: patients who did not experience complications requiring rehabilitation treatment (*n* = 37), 1 = Cases: patients who developed complications and underwent rehabilitation treatment (*n* = 34). Nonasymptomatic: *n* = 46/71. For recovery times: 0 = no recovery, 1 = within 3 months from the first physiotherapy visit, 2 = within 6 months from the first physiotherapy visit, 3 = after 6 months from the first physiotherapy visit. Chi‐square, T, and ANOVA tests were applied with significant values (*p* < 0.001). DASH, Disability of the Arm, Shoulder and Hand questionnaire.

**FIGURE 3:**
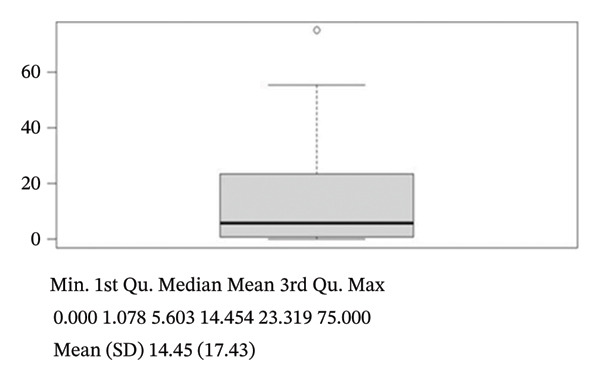
Boxplot 2 observing the interquartile range (IQR), the distribution of values appears asymmetric with a median value of 5.6 and an outlier recorded at 75. The mean of values was 14.45 (SD 17.43).

From the analysis of the DASH scores, we obtained an average questionnaire score of 14.45 (±17.43). Observing the interquartile range (IQR) of the boxplot, the distribution of values appeared to be asymmetric with a median value of 5.6 and an outlier that recorded a value of 75 (Figure 3: Boxplot 2). Furthermore, we evaluated the DASH scores in cases and controls, and the mean DASH values in the two groups were found to be statistically different (*p* ≤ 0.0001), with a higher mean value in cases compared to controls (Table 1).

Moreover, when evaluating the mean DASH scores in relation to recovery times, these were found to be statistically different, with an average DASH value of 50% in those who did not recover, and an average value of 35% in those who recovered after 6 months, compared to those who recovered within 3–6 months from the first physiatric visit (Table 2). Evaluating the average age of patients in relation to recovery and recovery times, this was found to be statistically different for both with the presence of recovery in older patients on average older (59.14 years) and who recovered in shorter times, for example, those with an average age of 61 years recovered within 3 months from the physiatric visit (*p* ≤ 0.05) (Table 3).

**TABLE 2:** Association of the DASH questionnaire with recovery times.

	Mean	Median	SD	*p*‐value
0	50.41	50.41	34.76	**0.05487**
1	15.41	12.50	13.09	
2	21.71	20.25	18.09	
3	35.25	37.06	15.84	

*Note:* 0 = no recovery, 1 = within 3 months from the first physiotherapy visit, 2 = within 6 months from the first physiotherapy visit. The ANOVA test was applied with significant values (*p* < 0.05) highlighted in bold.

**TABLE 3:** Evaluation of average age in relation to recovery, DASH, and recovery times.

Age	Recovery		DASH	Recovery times			
	No	Yes		0	1	2	3
	50.5	59.14	−0.176	50.5	61.35	57.00	59.36
*p*‐value	**0.009451**		0.143	**0.007929**			

*Note:* Recovery times: 0 = no recovery, 1 = within 3 months from the first physiatric visit. 2 = within 6 months from the first physiatric visit, 3 = after 6 months from the physiatric visit. Times are expressed as averages ± SD. The T and ANOVA tests were applied, with significant values (*p* < 0.05) highlighted in bold.

Finally, evaluating complications in relation to recovery and recovery times, there was a statistical association with respect to absent/present recovery for LE (*p* = 0.02113), DS (*p* = 0.0527), and SW (*p* = 0.02113); instead, in relation to recovery times, it was observed for LE (*p* = 0.0020), SPML (*p* ≤ 0.0001), DM (*p* = 0.0044), DS (*p* = 0.0220), and SW (*p* = 0.0510). Furthermore, the mean DASH scores were statistically different for LE (*p* = 0.0619), SPML (*p* ≤ 0.0001), DM (*p* ≤ 0.0001), DS (*p* = 0.0214), and SW (*p* = 0.0164), with higher DASH values in those who presented these complications.

## 4. Discussion and Conclusions

From the overall clinical characteristics of the sample under examination, it was observed that the most frequently developed complications were DM, DS, and SPML, which required treatment with a FKT‐based rehabilitation program. In our cohort, 34 of the 71 patients (47.89%) developed complications requiring rehabilitative treatment and were therefore classified as cases, whereas slightly more than half of the patients (*n* = 37/71) did not require specific postoperative rehabilitation among whom 25 remained completely asymptomatic and 12 developed self‐limiting complications.

In line with AIOM guidelines, a more advanced stage of disease was confirmed in those undergoing ALND, predominantly stage IIIA, compared to stage IA in those undergoing SLNB. Additionally, we verified that the number of lymph nodes removed increased depending on the type of axillary intervention, with an average of 13 lymph nodes for ALND and 3 for SLNB. This confirmed the appropriateness of lymph node sampling performed in the two surgical staging procedures of the axillary cavity.

We have also demonstrated that there is a higher average number of lymph nodes removed, about three times as many, in those who developed complications requiring rehabilitative intervention, and therefore, we can assert that this is a potential increased risk factor. Indeed, comparing cases and controls, proportionally more demolitive axillary interventions were observed in the case group, such as ALND, or repeated in the same site, such as ALND after SLNB. Therefore, more extensive surgical interventions were associated with a greater development of complications and the need for rehabilitative treatment. In particular, a significant correlation emerged with the specific complications DM and DS and the total number of lymph nodes removed, with mean values of approximately 8 total lymph nodes excised in subjects who presented these complications out of the total selected population.

We observed a statistically significant association between SC and BMI, with a higher incidence in patients with lower BMI. This finding is consistent with previous reports in the literature [34].

Evaluating specific complications in those who required rehabilitation treatment for this reason, AWS, in our case cohort, was observed more in those who underwent SLNB, with an incidence of 42%, compared to what is reported in the literature with a higher incidence after ALND [27]. This observation could, however, depend on an internal bias in the case group, namely the fact that the SLNB intervention (71.83%) prevailed over ALND (21.13%). To overcome this internal bias, future strategies to be applied include a more balanced initial selection among the types of axillary interventions.

**FIGURE 4:**
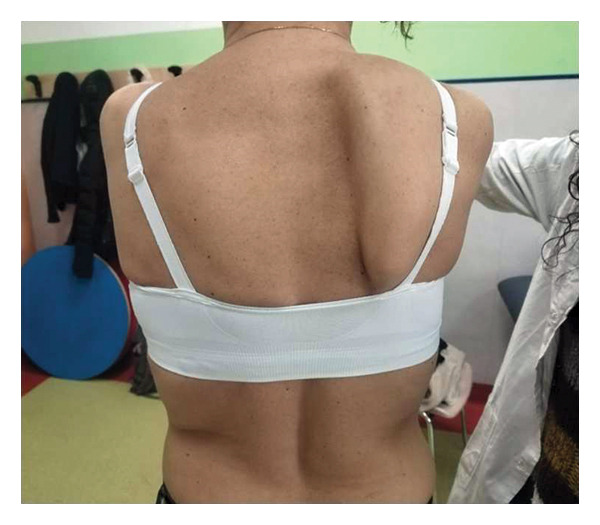
Representative case of postoperative scapular winging observed in our “case” cohort following ALND after positive SLNB. ALND = axillary lymph node dissection. SLNB = axillary sentinel lymph node biopsy.

Another significant element that emerged from our analysis of the case group was the very low percentage (4.32%) of onset of more disabling complications with a greater impact on QoL, such as LE and SW; a representative clinical example is shown in Figure 4. These data confirmed that BC care should be centralized in Breast Units where proper surgical techniques can minimize postoperative axillary complications. Notably, SW formation rates after surgery could vary unexplainably widely from 0% to 74.7% [29].

Regarding recovery, among the symptomatic patients who developed postoperative complications (*n* = 46), recovery was observed in 91.3%, suggesting that postoperative functional impairments may improve either spontaneously (*n* = 12) or with targeted rehabilitative management (*n* = 34, cases).

In particular, in patients presenting minor complications, such as DS, DM, and SPML, we observed recovery within a shorter time frame, specifically within 3 months from the first physiatric visit; instead, in line with the literature, in most patients who presented complications, such as LE and SW, we did not find a recovery (86% in our case group) and any achievement of it resulted in requiring longer treatment times, exceeding 6 months from the first physiatric visit [30].

However, overall patients in the case group showed a higher rate of recovery compared with controls. This may reflect the effect of targeted rehabilitative interventions, which likely contributed significantly to functional improvement in patients presenting more postoperative complications.

In line with this, the average DASH score was 14%, indicating an overall low level of disability among the study population within the 0%–100% scale. Worse patient‐reported outcomes (PROs) measured by the DASH questionnaire were mainly observed in patients who developed postoperative complications requiring rehabilitative treatment and in those with delayed, beyond 6 months, or absent recovery. In particular, significantly higher DASH scores were associated with postoperative complications, SPML, DM, DS, and SW. These findings highlight the impact of postoperative functional complications on PROs and support the usefulness of the DASH questionnaire in detecting clinically relevant upper limb disability after axillary surgery. The mandatory main module of DASH was used because, through 30 questions, it provides a comprehensive and validated assessment of upper limb functionality, covering all relevant daily aspects of possible disability experienced. Instead, the supplementary modules focus on specific activities, such as sports or music, which are not practiced by the entire study population. In fact, using only the main module allows for standardization of DASH values, considering that optional modules may not be completed by all patients. The application of telephone self‐assessment presents significant advantages, such as greater accessibility in questionnaire administration, reaching even patients with limited mobility and transportation difficulties, with increased patient compliance. However, it should be emphasized that this approach also involves some limitations, including the difficulty in ensuring that all patients fully understand all questions and the impossibility of concomitant clinical observation. However, the problems detected by the questionnaire and perceived by the patient are an important point of discussion for subsequent surgical or physiatric follow‐up visits.

Contrary to expectations, younger patients on average appeared to develop more complications, such as DS and DM, than older women, with a consequent greater need for rehabilitation treatment. Indeed, we observed a quicker recovery from complications in older patients. External factors to surgery, such as lifestyle, work activity, and more active social–relational life in younger women, likely lead to greater functional limitations that require more time for eventual recovery.

In conclusion, although major complications after axillary surgery are relatively uncommon [35], functional impairment of the upper limb remains frequent and may significantly affect patients’ daily activities. These findings support the growing interest in de‐escalation strategies in axillary surgery, aiming to limit the use of ALND and favor more conservative and selective staging approaches whenever oncologically appropriate, both in the upfront surgical and neoadjuvant settings [6, 37, 38]. In this context, integrating surgical management within the Breast Unit together with early physiatric evaluation and rehabilitation plays an important role in optimizing functional recovery after BC surgery. Systematic functional assessment using patient‐reported outcome measures (PROMs), such as the DASH questionnaire, may therefore represent a valuable tool for early identification of disability and for the longitudinal follow‐up of patients. Further research may help refine therapeutic strategies and improve patient QoL.

## Author Contributions

Monica Pellegrino: conceptualization, project administration, data curation, software, formal analysis, visualization, writing–original draft, and writing–review and editing. Francesco Ioppolo: project administration, supervision, formal analysis, and writing–review and editing. Monia Ranalli: methodology, supervision, formal analysis, software, writing–original draft, and writing–review and editing. Michelangelo Miccini: conceptualization, methodology, and supervision. Francesca La Rovere: data curation, formal analysis, and writing–review and editing. Stefania Lancia: data curation, formal analysis, and writing–review and editing. Stefano Avenia: formal analysis and writing–review and editing. Paolo Izzo: data curation and writing–review and editing. Giulia Montagner: data curation and writing–review and editing. Giuliano D’Onghia: supervision, data curation, and writing–review and editing. Andrea Polistena: conceptualization, supervision, project administration, validation, and writing–review and editing.

## Funding

No funding was received for this manuscript. Open access publishing facilitated by Universita degli Studi di Roma La Sapienza, as part of the Wiley ‐ CRUI‐CARE agreement.

## Disclosure

The authors are accountable for all aspects of the work in ensuring that questions related to the accuracy or integrity of any part of the work are appropriately investigated and resolved. All authors have read and approved the final version of the manuscript. *G. D’Onghia* had full access to all of the data in this study and takes complete responsibility for the integrity of the data and the accuracy of the data analysis.

## Ethics Statement

The study was conducted in accordance with the Declaration of Helsinki, but the protocol was not submitted to the evaluation of the local ethics committee or registered as a clinical trial due to the observational and retrospective design of the research. All patients gave their informed consent to the use of their clinical data for research purposes at the time of surgery.

## Conflicts of Interest

The authors declare no conflicts of interest.

## Supporting Information

Additional supporting information can be found online in the Supporting Information section.

## Supporting information


**Supporting Information** The supporting information includes the STROBE checklist, which was completed to ensure transparent reporting of this observational study. The checklist provides details on the reporting items addressed throughout the manuscript.

## Data Availability

The authors confirm that the relevant data supporting the findings of this study are presented within the article. Additional anonymized data underlying the results are available from the corresponding author upon reasonable request.

## References

[1] I Numeri Del Cancro in Italia 2025, AIOM-AIRTUM».

[2] Curigliano G. , Burstein H. , Gnant M. et al., Understanding Breast Cancer Complexity to Improve Patient Outcomes: The St Gallen International Consensus Conference for the Primary Therapy of Individuals With Early Breast Cancer 2023, Annals of Oncology. (2023) 34, no. 11, 970–986, 10.1016/j.annonc.2023.08.017. 37683978

[3] 19th St. Gallen Breast Cancer Conference March 2025–Vienna», 2025, SGBCC, https://www.sg-bcc.org/.

[4] Pia Schieroni M. , La Riabilitazione in Oncologia, Edizioni Medico Scientifiche, 2017, C.G. Edizioni Me dico Scientifiche.

[5] Associazione Italiana di Oncologia Medica (Aiom) , Linee Guida Carcinoma Mammario in Stadio Precoce, 2023, AIOM, https://www.aiom.it/linee-guida-aiom-2023-carcinoma-mammario-in-stadio-precoce/.

[6] Tinterri C. , Canavese G. , Gatzemeier W. et al., Sentinel Lymph Node Biopsy Versus Axillary Lymph Node Dissection in Breast Cancer Patients Undergoing Mastectomy With One to Two Metastatic Sentinel Lymph Nodes: Sub-Analysis of the SINODAR-ONE Multicentre Randomized Clinical Trial and Reopening of Enrolment, British Journal of Surgery. (2023) 110, no. 9, 1143–1152, 10.1093/bjs/znad215. 37471574 PMC10492188

[7] Goyal A. , Mann G. B. , Fallowfield L. et al., POSNOC—Positive Sentinel Node: Adjuvant Therapy Alone Versus Adjuvant Therapy Plus Clearance or Axillary Radiotherapy: A Randomised Controlled Trial of Axillary Treatment in Women With Early-Stage Breast Cancer Who Have Metastases in One or Two Sentinel Nodes, BMJ Open. (2021) 11, no. 12, 10.1136/bmjopen-2021-054365. PMC8640630 34857578

[8] «Guidelines Detail», NCCN, https://www.nccn.org/guidelines/guidelines-detail?category=1%26;id=1419.

[9] Associazione Nazionale Italiana Senologi Chirurghi (Anisc) , Linee Guida ANISC, 2026, ANISC, https://www.anisc.org/formazione-e-progetti/linee-guida-anisc/.

[10] Biganzoli L. , Cardoso F. , Beishon M. et al., The Requirements of a Specialist Breast Centre, Breast. (2020) 51, 65–84, 10.1016/j.breast.2020.02.003. 32217457 PMC7375681

[11] von Elm E. , Altman D. G. , Egger M. , Pocock S. J. , Gøtzsche P. C. , and Vandenbroucke J. P. , The Strengthening the Reporting of Observational Studies in Epidemiology (STROBE) Statement: Guidelines for Reporting Observational Studies, International Journal of Surgery. (2014) 12, no. 12, 1495–1499, 10.1016/j.ijsu.2014.07.013. 25046131

[12] Padua R. , Padua L. , Ceccarelli E. et al., Italian Version of the Disability of the Arm, Shoulder and Hand (DASH) Questionnaire. Cross-Cultural Adaptation and Validation, Journal of Hand Surgery (Edinburgh, Scotland). (April 2003) 28, no. 2, 179–186, 10.1016/s0266-7681(02)00303-0. 12631494

[13] Harrington S. , Michener L. A. , Kendig T. , Miale S. , and George e S. Z. , Patient-Reported Upper Extremity Outcome Measures Used in Breast Cancer Survivors: A Systematic Review, Archives of Physical Medicine and Rehabilitation. (2014) 95, no. 1, 153–162, 10.1016/j.apmr.2013.07.022. 23932969 PMC4162515

[14] Singh R. , Deo S. V. S. , Dhamija E. , Mathur S. , and Thulkar e S. , To Evaluate the Accuracy of Axillary Staging Using Ultrasound and Ultrasound-Guided Fine-Needle Aspiration Cytology (USG-FNAC) in Early Breast Cancer Patients—A Prospective Study, Indian Journal of Surgical Oncology. (2020) 11, no. 4, 726–734, 10.1007/s13193-020-01222-3. 33281412 PMC7714810

[15] Banys-Paluchowski M. , Gasparri M. , de Boniface J. et al., Surgical Management of the Axilla in Clinically Node-Positive Breast Cancer Patients Converting to Clinical Node Negativity Through Neoadjuvant Chemotherapy: Current Status, Knowledge Gaps, and Rationale for the EUBREAST-03 AXSANA Study, Cancers. (2021) 13, no. 7, 10.3390/cancers13071565. PMC8037995 33805367

[16] «Hartmann S. , Kühn T. , Hauptmann M. et al., Axillary Staging After Neoadjuvant Chemotherapy for Initially Node-Positive Breast Carcinoma in Germany: Initial Data From the AXSANA Study, Geburtshilfe Und Frauenheilkunde. (September 13 2022) 82, no. 9, 932–940, 10.1055/a-1889-7883. 36110892 PMC9470287

[17] «Axillary Surgery After NeoAdjuvant Treatment (Axsana) , Clinicaltrials.Gov, 2025, https://www.clinicaltrials.gov/study/NCT04373655.

[18] «Kühn T. , Banys-Paluchowski M. , Ditsch N. et al., More Versus Less Invasive Axillary Surgical Staging Procedures in Breast Cancer Patients Converting From a Clinically Node-Positive to a Clinically Node-Negative Stage Through Neoadjuvant Chemotherapy–Primary Endpoint Analysis of the International Prospective Multicenter AXSANA/EUBREAST 3(R)Study, Clinical Cancer Research. (2025) 32, no. 4_Supplement, San Antonio, Texas, Presented at: 2025 San Antonio Breast Cancer Symposium 10.1158/1557-3265.SABCS25-GS2-01.

[19] Mhs A. E. , «Less Invasive Axillary Staging is Noninferior to ALND in Post-NACT Node-Negative Breast Cancer, Targeted Oncology–Immunotherapy, Biomarkers, and Cancer Pathways», Dic. (2025) https://www.targetedonc.com/view/less-invasive-axillary-staging-is-noninferior-to-alnd-in-post-nact-node-negative-breast-cancer.

[20] Scuola Italiana di Senologia Onlus , Linfonodo Sentinella. Scuola Italiana Di Senologia ONLUS, 2026, https://www.senologia.it/la-biopsia-del-linfonodo-sentinella/.

[21] Rubio I. , Pedreira F. , Roca I. et al., Removal of all Radioactive Sentinel Nodes in Breast Cancer Improves the Detection of Positive Sentinel Nodes, Clinical and Translational Oncology. (2008) 10, no. 6, 347–350, 10.1007/s12094-008-0210-0. 18558581

[22] Sanguinetti A. , Bistoni G. , and Avenia N. , Chirurgia Della Mammella: Testo-Atlante, Terni: Morphema Editrice. (2010) .

[23] SiapeC-Iap , GIPAM Recommendations on Sentinel Lymph Node, 2021, SIAPeC-IAP, https://www.siapec.it/2021/11/10/raccomandazioni-gipam-sul-linfonodo-sentinella/.

[24] Italian Association of Medical Oncology (Aiom) , Linee Guida Neoplasie Della Mammella, 2021, AIOM, https://www.aiom.it/linee-guida-aiom-2021-neoplasie-della-mammella/. 10.1177/030089160409000229 15237598

[25] de Oliveira J. F. et al., Incidence and Risk Factors of Winged Scapula After Axillary Lymph Node Dissection in Breast Cancer Surgery, Applied Cancer Research. (2009) 29, no. 2.

[26] Koehler L. A. , Haddad T. C. , Hunter D. W. , and Tuttle e T. M. , Axillary Web Syndrome Following Breast Cancer Surgery: Symptoms, Complications, and Management Strategies, Breast Cancer Dove Medical Press. (2019) 11, 13–19, 10.2147/BCTT.S146635. 30588087 PMC6304256

[27] Moskovitz A. H. , Anderson B. O. , Yeung R. S. , Byrd D. R. , Lawton T. J. , and Moe e R. E. , Axillary Web Syndrome After Axillary Dissection, Americas Journal of Surgery. (2001) 181, no. 5, 434–439, 10.1016/s0002-9610(01)00602-x. 11448437

[28] Ferrandez J.-C. , Cinesiterapia Dopo Cancro Del Seno, EMC–Medical Rehabilitation. (2010) 17, no. 4, 1–13, 10.1016/S1283-078X(10)70198-X.

[29] Adriaenssens N. , De Ridder M. , Lievens P. et al., Scapula Alata in Early Breast Cancer Patients Enrolled in a Randomized Clinical Trial of Post-Surgery Short-Course Image-Guided Radiotherapy, World Journal of Surgical Oncology. (2012) 10, no. 1, 10.1186/1477-7819-10-86. PMC3488523 22591589

[30] Schieroni M. P. , Valobra G. N. , Gatto R. , and Monticone M. , Clinical and Rehabilitative Approach to Patients Operated on for Breast Cancer, Nuovo Trattato Di Medicina Fisica e Riabilitazione, 2009, UTET, Turin.

[31] Mammucari M. , Maggiori E. , Russo D. et al., Mesotherapy: From Historical Notes to Scientific Evidence and Future Prospects, Scientific World Journal. (2020) 2020, 3542848–3542849, 10.1155/2020/3542848. 32577099 PMC7305548

[32] Complex Decongestive Lymphatic Therapy With or Without Vodder II Manual Lymph Drainage in More Severe Chronic Postmastectomy Upper Limb Lymphedema: A Randomized Noninferiority Prospective Study–Pubmed, https://pubmed.ncbi.nlm.nih.gov/26303187/. 10.1016/j.jpainsymman.2015.06.017 26303187

[33] Jester A. , Harth A. , Wind G. , Germann G. , and Sauerbier e M. , Disabilities of the Arm, Shoulder and Hand (DASH) Questionnaire: Determining Functional Activity Profiles in Patients With Upper Extremity Disorders, Journal of Hand Surgery (European Volume). (2005) 30, no. 1, 23–28, 10.1016/j.jhsb.2004.08.008. 15620487

[34] Bergmann A. , Mendes V. V. , de Almeida Dias R. , do Amaral E Silva B. , da Costa Leite Ferreira M. G. , and Fabro e E. A. N. , Incidence and Risk Factors for Axillary Web Syndrome After Breast Cancer Surgery, Breast Cancer Research and Treatment. (2012) 131, no. 3, 987–992, 10.1007/s10549-011-1805-7. 21987036

[35] Al-Hilli Z. and Wilkerson A. , Breast Surgery: Management of Postoperative Complications Following Operations for Breast Cancer, Surgical Clinics of North America. (2021) 101, no. 5, 845–863, 10.1016/j.suc.2021.06.014. 34537147

[36] Giuliano A. E. , Ballman K. V. , McCall L. et al., Effect of Axillary Dissection vs No Axillary Dissection on 10-Year Overall Survival Among Women With Invasive Breast Cancer and Sentinel Node Metastasis: The ACOSOG Z0011 (Alliance) Randomized Clinical Trial, JAMA. (2017) 318, no. 10, 918–926, 10.1001/jama.2017.11470. 28898379 PMC5672806

[37] Different Strategies in De-Escalation of Axillary Surgery in Node-Positive Breast Cancer Following Neoadjuvant Treatment: A Systematic Review and Meta-Analysis of Long-Term Outcomes–Pubmed, https://pubmed.ncbi.nlm.nih.gov/40186790/. 10.1007/s12282-025-01692-9 PMC12174186 40186790

[38] Vieira R. A. da C. , da Costa A. M. , de Souza J. L. et al., Risk Factors for Arm Lymphedema in a Cohort of Breast Cancer Patients Followed up for 10 Years, Breast Care Basel Switzerland. (2016) 11, no. 1, 45–50, 10.1159/000442489. 27051396 PMC4813649

